# An Image-Recognition Dietary Assessment App for Adolescents With Obesity: Pilot Randomized Controlled Trial

**DOI:** 10.2196/58682

**Published:** 2024-12-02

**Authors:** Krista Oei, Elizabeth EY Choi, Alisa Bar-Dayan, Jennifer N Stinson, Mark R Palmert, Jeffrey E Alfonsi, Jill Hamilton

**Affiliations:** 1 Division of Endocrinology The Hospital for Sick Children Toronto, ON Canada; 2 Department of Pediatrics University of Toronto Toronto, ON Canada; 3 Research Department RxFood Corporation Toronto, ON Canada; 4 Clinical Dietetics The Hospital for Sick Children Toronto, ON Canada; 5 Research Institute The Hospital for Sick Children Toronto, ON Canada; 6 Lawrence S Bloomberg Faculty of Nursing University of Toronto Toronto, ON Canada; 7 Department of Physiology University of Toronto Toronto, ON Canada; 8 Department of Medicine University of Toronto Toronto, ON Canada; 9 Department of Nutritional Sciences University of Toronto Toronto, ON Canada

**Keywords:** obesity, weight management, dietary assessment, nutrition, adolescent, digital health app, mHealth, mobile health, image recognition, teens, weight, youth, diet, dietary, dietary app, usability, feasibility, pilot randomized controlled trial, obesity management, nutritional, user, patient engagement, mobile phone

## Abstract

**Background:**

Adolescence is a period of increased susceptibility to developing obesity-related health issues due to poor eating patterns and increased sedentary behaviors. Recommendations for pediatric obesity management include dietary assessments. However, adolescents often avoid food logging through traditional methods. The use of image-recognition dietary assessment apps in adolescents with obesity is not well studied. Eating for Wellness (E4W) is a mobile app that determines the nutritional content of meals from photos and incorporates nutritional goal setting. Nutritional data can be displayed for health care providers (HCPs) via the Clinician Portal, while the data are presented to the user in a manner that minimizes the focus on calorie counting.

**Objective:**

This study aims to evaluate the usability and feasibility of E4W, a mobile health app designed to improve dietary intake in adolescents with obesity attending an obesity clinic, using a phased approach.

**Methods:**

The overall study was conducted in 2 phases to refine and evaluate E4W. In Phase 1, usability was tested through 3 iterative cycles of patient interviews. A total of 14 patient participants, aged 12-18 years with a BMI≥97th percentile, were included. Participants performed standardized scenario-based tasks in E4W and provided feedback on the app. Two iterative cycles were conducted for HCPs (n=4). Refinements were made during each cycle based on issues encountered and feedback provided. In Phase 2, a pilot randomized controlled trial of 32 adolescents (16 adolescents enrolled in the experimental group for 1 month, and 16 controls enrolled for 1 month) was completed. Both groups met with their dietitian at baseline, midstudy, and 1 month following their baseline visit to discuss goals and eating patterns. The control group was instructed to take photos of all intake using their default phone camera, without access to E4W, while those in the experimental group received full access to E4W. The primary outcome was the feasibility of implementation. Secondary outcomes examined overall change in dietary intake and achievement of nutritional goals.

**Results:**

Usability testing demonstrated that E4W and the Clinician Portal were easy to use, efficient, and well-liked by patients and HCPs. Feasibility testing revealed high patient acceptability scores. However, significant technical challenges were encountered. Although the use of E4W did not significantly impact patient engagement (control: mean 0.9, SD 0.7; experimental: mean 1.7, SD 1.9; *P*=.14), there were outliers in the experimental group with very high engagement and improved self-reported efficacy. Overall, there was no improvement in dietary intake, although assessment was hindered by poor adherence to traditional methods of food logging.

**Conclusions:**

E4W and the Clinician Portal were well-received by patients and HCPs. Further research is warranted and planned to determine if E4W can improve dietary intake and achievement of nutritional goals in adolescents with obesity.

**Trial Registration:**

ClinicalTrials.gov NCT05548868; https://clinicaltrials.gov/study/NCT05548868

## Introduction

Adolescence is currently a period of increased susceptibility for developing obesity-related health issues, concordant with poor eating patterns and increased sedentary behaviors [1]. Best practice recommendations for pediatric obesity management include a family-based, multifaceted, interdisciplinary approach with frequent contact between patients and health care providers (HCPs) [2] to promote gradual behavioral changes, including improved eating patterns and diet quality [3,4]. Registered dietitians assess food intake using various methods such as written food records or dietary recall. Unfortunately, these traditional methods are often hindered by low participant engagement [5], inaccuracy [6], poor reliability, and lengthy completion time [7]. Studies on children with obesity have shown that their self-reported caloric intake is either equal [8] or lower [9] compared to their normal-weight peers. It is unclear whether this is due to errors in study design, social pressures to underreport, or perhaps difficulties estimating portion sizes. Since adolescents have greater independence in regard to their dietary choices [10], efforts to promote healthy eating patterns must include engaging, developmentally appropriate, accurate, and easy-to-use methods of dietary assessment.

Image-based mobile apps, which allow users to photograph their meals, are emerging as sophisticated tools for assessing intake that can address some of the limitations of traditional methods and may improve self-management of intake [6,11]. Mobile devices are now equipped with high-quality cameras, high-speed processors, and wireless connectivity, which make them ideal for capturing intake. Since dietary intake methods are prone to errors [12], these new advancements in technology can help improve the accuracy of dietitians to effectively counsel patients on their eating patterns. However, few studies have examined image-based mobile apps for dietary assessment in children and adolescents [13-17].

Although there are many nutrition and weight management apps on the market, many include an emphasis on calorie counting for weight loss [18], which can be problematic for adolescents with obesity who are at increased risk of developing eating disorders. To address these concerns, Eating for Wellness (E4W) was developed as a tool to be used in pediatric weight management programs for improved assessment of dietary intake and to increase program engagement through increased contact with HCPs. E4W is an image-recognition app that uses proprietary algorithms to analyze photographs of meals and snacks to determine their nutritional components. The nutritional intake is displayed in a way that minimizes the focus on calorie counting, with detailed nutritional components sent directly to the dietitian via the Clinician Portal without being visible to the patient. With E4W, adolescents can participate in goal setting and receive timely feedback and personalized self-monitoring support from their dietitian.

This study aimed to (1) complete usability testing of E4W and the Clinician Portal and refine it for use in pediatric weight management programs and then (2) determine whether E4W and the Clinician Portal could be feasibly implemented. We hypothesized that E4W would be user-friendly and accepted by adolescents receiving care in a pediatric weight management program and that it could be feasibly implemented.

## Methods

### E4W Design

**Figure 1:**
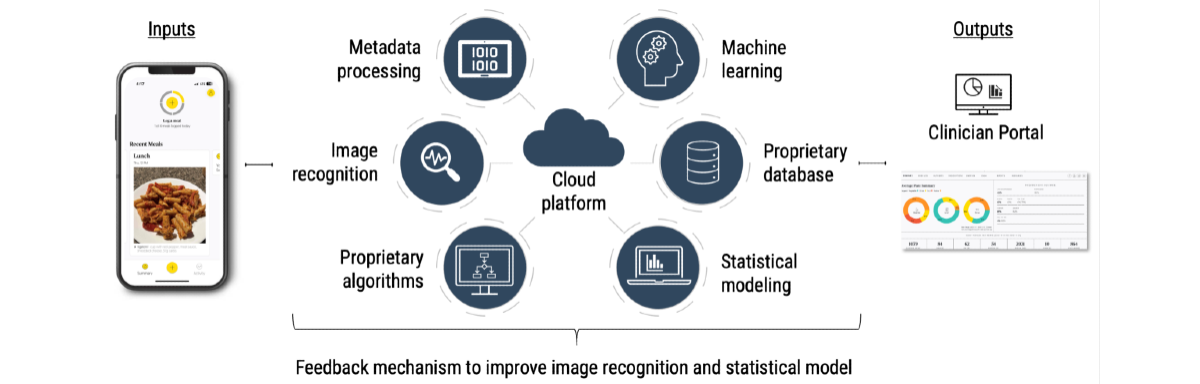
E4W system architecture. Patient takes photos of a meal with their smartphone. Data are transmitted to the cloud for analysis. Identified food and portion sizes are transmitted to the app (adolescent). Detailed nutrient profile is sent to the secure Clinician Portal (dietitian). E4W: Eating for Wellness.

E4W uses existing proprietary algorithms (Figure 1) from iSpy [19] and RxFood [20]. The backend infrastructure is driven by several core components. When a photo of a meal is taken, it is inputted into the analysis engine, which uses context and statistical analysis to work alongside a convolutional neural network–driven computer vision module to obtain a list of the most probable foods. This output of foods then works with the proprietary data structure to obtain nutrient profiles (based on the Canadian Nutrient File, US Department of Agriculture National Nutrient Database, and information directly from a manufacturer), volumes, etc, to provide feedback to the algorithm to further enhance accuracy. The user can then confirm that the item identified is accurate. The nutritional data are analyzed and displayed for the clinician via the Clinician Portal, while the data are presented to the user in a manner that minimizes the focus on calorie counting. The app’s proprietary technology uses the recognition of nearby objects and triangulation of multiple images using bursts of photos or multiple cameras to improve portion estimates. This eliminates the need for fiducial markers and reduces participant burden. If the meal is not finished, the user can use a slider button to enter the percentage eaten within the user interface. This then updates the portion size of the food item, allowing for accountability of plate waste along with better estimation of nutrients consumed. E4W was also customized to include tailored self-monitoring support via personalized messages from HCPs based on the adolescent’s eating patterns and goals. Other automated push notifications, positive messaging, and reminders are sent by the app. Goal-setting features were added to provide coaching in line with the healthy plate model [21].

### Phased Approach to Refining E4W

The overall study was conducted using a phased approach to evaluate and refine E4W. Phase-1 usability testing adopted a patient-centered approach to establish whether the app was viable (user-friendly and acceptable) and refine it to be ready for use in Phase 2. Phase-2 feasibility testing was designed to establish E4W’s feasibility in a pilot randomized controlled trial (RCT).

### Study Population

Data were collected from study participants enrolled in the Hospital for Sick Children’s Healthy Living Clinic (HLC) and interdisciplinary health care team members working in HLC. Patient inclusion criteria were (1) aged 12-18 years, (2) BMI≥97th percentile with significant weight-related comorbidities or BMI≥99th percentile, (3) English speaking, and (4) smartphone ownership. Adolescents with severe cognitive impairments, an unstable eating disorder, unstable mental health, or a history of bariatric surgery were excluded from the study.

### Study Design and Procedures

#### Phase-1 Usability Testing

Qualitative usability testing consisted of semistructured, audiotaped interviews and user observation with iterative cycles. Each cycle consisted of 6 task-based scenarios that were developed using standardized guidelines [22] and evaluated for likes and dislikes, satisfaction, and suggestions for improvement through semistructured interviews and completion of Acceptability E-Scale (AES) questionnaires [23,24].

Based on recommendations [25] and previous team experience [26], each usability cycle included 4-6 adolescents and 2 HCPs, with 2-3 cycles to identify and resolve issues [19,27,28]. HLC patients who met inclusion criteria received a study invitation from an HLC team member on behalf of the study principal investigator. Patients were then contacted via email or approached in person at routine clinical visits to explain the study and written consent was obtained for those interested in participating. Patients and HCPs were purposively selected to achieve diversity in age, sex, and duration in HLC patients and diversity in health care roles [26]. Errors and efficiency (the time it took to complete a task) were logged. Difficulties completing a task (ease of use) and any suggestions and preferences were also noted. Completion of tasks was graded as either successfully completed, completed with minor issues, or incomplete due to critical usability issues. Tasks in each category were totaled and the mean number of incomplete tasks due to a critical issue per participant was calculated. Following each cycle, updates to E4W and the Clinician Portal were completed based on errors identified and improvements suggested from the usability testing and interview content analysis. The revised app was then used in the next cycle [27,29] and then iteratively evaluated until no further problems were found [19,27,28]. Interviews and testing were conducted virtually using a secure teleconferencing platform (Microsoft Teams). This iterative design approach focused on usability criteria (ease of use, learnability, errors, and efficiency) and satisfaction with content and functionality. Questionnaire results of AES were analyzed and presented as mean (SD).

#### Phase-1 Data Analysis

Demographics were analyzed using descriptive statistics. Categorical variables were described as frequency and percentages. Continuous data was presented as mean (SD). Excel 365 (version 2304; Microsoft) and SPSS Statistics (version 29.0.1.0(171); IBM) were used for all statistical analyses.

Audiotaped interviews and field notes were transcribed and used in simple descriptive qualitative content analysis [30]. The data were managed using Excel 365. Data analyses began after the first interview to engage an iterative process such that errors and suggestions for improvement highlighted in earlier interviews could inform subsequent interviews (using constant comparative analyses) [31]. Themes were generated from the data, and categories were identified within the themes, which highlighted specific items that needed to be fixed and other patient suggestions related to content, aesthetics, functionality, and usability. As data were analyzed, categories continued to be generated until there were no new data to be categorized (ie, saturation) [32].

#### Phase-2 Feasibility Testing

##### Overview

Phase 2 was designed as an open-label, parallel-group, pilot RCT consistent with CONSORT-C (Consolidated Standards of Reporting Trials–Children) and SPIRIT-C (Standard Protocol Items for Randomized Trials–Children) recommendations for designing, conducting, and reporting pediatric clinical trials [33,34]. HLC patients who met inclusion criteria received a study invitation from an HLC team member on behalf of the study principal investigator. Patients were then contacted via email or approached in person at routine clinical visits to explain the study and written consent was obtained for those interested in participating. A convenience sample of 32 adolescents in total was recruited as per pilot study recommendations of 10-20 participants per group [35-37] and to account for any potential loss to follow up while examining primary and secondary outcomes. RStudio (version 2021.09.2+382, PBC) [38] was used to allocate participants to control or experimental groups. There was equal allocation between groups (control: 16 participants; experimental: 16 participants).

Participants in both groups were asked to complete a brief web-based demographic questionnaire via a secure REDCap (Research Electronic Data Capture; Vanderbilt University) link. Additionally, baseline descriptive data were extracted from their electronic medical record.

The duration of the treatment period was approximately 4 weeks with dietitian assessments at baseline (visit 1), midstudy (visit 2, approximately week 2), and at the final visit (visit 3, approximately end of week 4). As per standardized care, dietitians incorporated SMART (Specific Measurable Attainable Realistic Time-Bound) goals into their visits. To standardize goal setting across both groups, a minimum of 3 goals were set at each visit. Attainment of goals for all participants was tracked manually through chart review. Participants in both groups were instructed to complete manual 3-day food records and take photos of their meals for their visit 1 and visit 3, and these were entered into the Automated Self-Administered 24 Hour Dietary Assessment Tool (ASA24; ASA24 Canada 2018, version released in April 2019 [39], a clinical standard for dietary assessment [40]) to determine change in dietary intake. Daily food records with <1000 kcal were removed from the analysis to remove implausibly low daily energy intake due to missing or inaccurate data.

##### Control Group Only

Adolescents in the control group were instructed to take photos of all meals and snacks, including beverages, using their default phone camera, without access to E4W. Participants also received weekly reminder emails from the research team reminding them to log their food intake. This design was implemented to ensure technology use and weekly contact in both arms for comparative purposes and to control for the possible effects of time and the impact of general smartphone use during the study period [41].

##### Experimental Group Only

Adolescents in the experimental group downloaded the E4W app onto their iOS or Android personal smartphones. Participants were instructed to take photos of all meals and snacks, including beverages, during the study period. After the photos were taken, the nutritional content was automatically analyzed and immediately sent to the Clinician Portal to be available for viewing by the dietitians. Dietitians used the following three-step framework to ensure consistency in feedback provided via the app: (1) three automatic reminder notifications to log food intake were sent per day during a 3-day period prior to visit 2 and visit 3 appointments, (2) personalized tips were sent by the dietitians once a week via the app, and (3) the dietitians used the Clinician Portal to access reports and detailed dietary information in preparation for study visits.

#### Phase-2 Data Analysis

Baseline demographics, clinical characteristics, and technology use were analyzed using descriptive statistics. Excel 365 and SPSS Statistics were used for all statistical analyses.

Quantitative data on completion rates, technical difficulties, acceptability and satisfaction, and engagement were analyzed using descriptive statistics. Qualitative data on types of technical difficulties encountered were also logged. Initial criteria for implementation success were based on feasibility studies previously conducted by Stinson et al [42] and included study completion rates >70%, minimal technical difficulties (eg, reported by <10%), high acceptability and satisfaction (mean score of ≥4 out of 5 on AES), and patient engagement and adherence rates (eg, >80% completion of food documenting and photo taking). After the intervention, qualitative interviews were conducted with the adolescents for overall feedback and suggestions for improvement of the app.

An exploratory analysis of the differences between the experimental group and control group was conducted using 2-sided independent *t* tests for continuous variables. While it was recognized that the nature of the pilot trial renders it underpowered to detect differences in dietary intake, such a preliminary analysis was performed to determine its feasibility and to determine the standard deviation and effect size of the sample for future studies.

### Setting

All visits and interviews were performed either in person at the Hospital for Sick Children or virtually via Microsoft Teams or Zoom (Zoom Video Communications, Inc) between February 2022 and April 2023.

### Ethical Considerations

This study was approved by the Research Ethics Board of the Hospital for Sick Children in Toronto (Phase 1 Usability Testing: 1000077293; Phase 2 Feasibility Testing: 1000079525). All patients and HCPs provided their informed consent to participate in this study. All data collected were anonymized, and data were collected and managed using REDCap electronic data capture tools hosted at The Hospital for Sick Children [43,44]. Phase-2 feasibility testing RCT was registered with ClinicalTrials.gov (NCT05548868).

## Results

### Phase 1—Usability Testing

#### Patient and HCP Characteristics

Patient and HCP usability testing and interviews were conducted virtually using Microsoft Teams between February and June 2022. Adolescents aged 12-18 years took part in the usability testing across 3 cycles (total of 14 participants; cycle 1: n=6, cycle 2: n=4, and cycle 3: n=4; Table 1). A total of 4 HCPs participated in 2 cycles (cycle 1: one registered dietitian and 1 nurse practitioner; cycle 2: one registered nurse and 1 exercise therapist) of usability testing of the Clinician Portal and interviews.

**Table 1:** Phase 1 usability testing—patient demographic characteristics.

			All participants (3 cycles; n=14)
**Age (years),** **mean (SD)**			15.2 (2.1)
**Sex, n (%)**			
	Male	8 (57)	
	Female	5 (36)	
	Nonbinary	1 (7)	
**Ethnicity, n (%)**			
	Asian	6 (43)	
	Black	2 (14)	
	White	5 (36)	
	Other	1 (7)	
**Time in HLC^a^** **(years),** **mean (SD)**			1.7 (1.2)
**Type of phone, n (%)**			
	Android	6 (43)	
	iPhone	8 (57)	

^a^HLC: Healthy Living Clinic.

#### Patient Usability Testing

**Figure 2:**
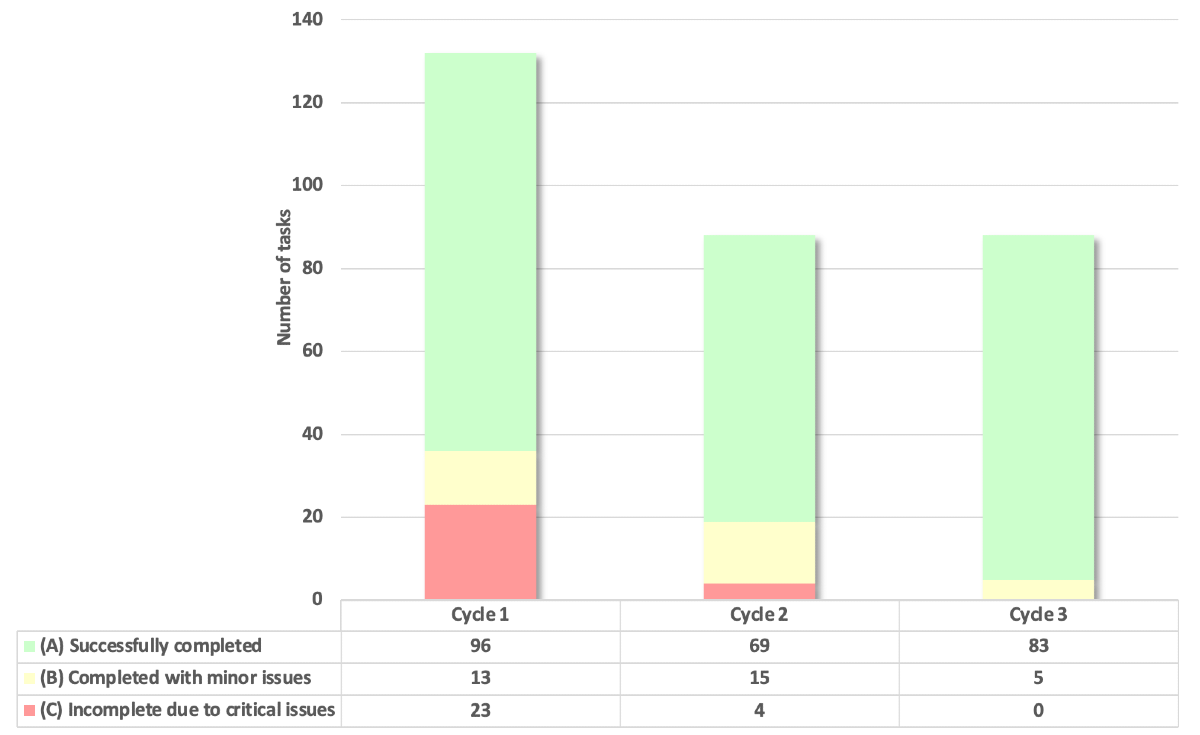
Phase-1 usability testing—patient results. Errors and issues were tracked for each cycle and completion of tasks was classified into 1 of 3 categories: (A) successfully completed (shown in green), (B) completed with minor issues (shown in yellow), and (C) incomplete due to critical usability issues (shown in red). The figure shows the total tasks per category for the 3 cycles.

**Figure 3:**
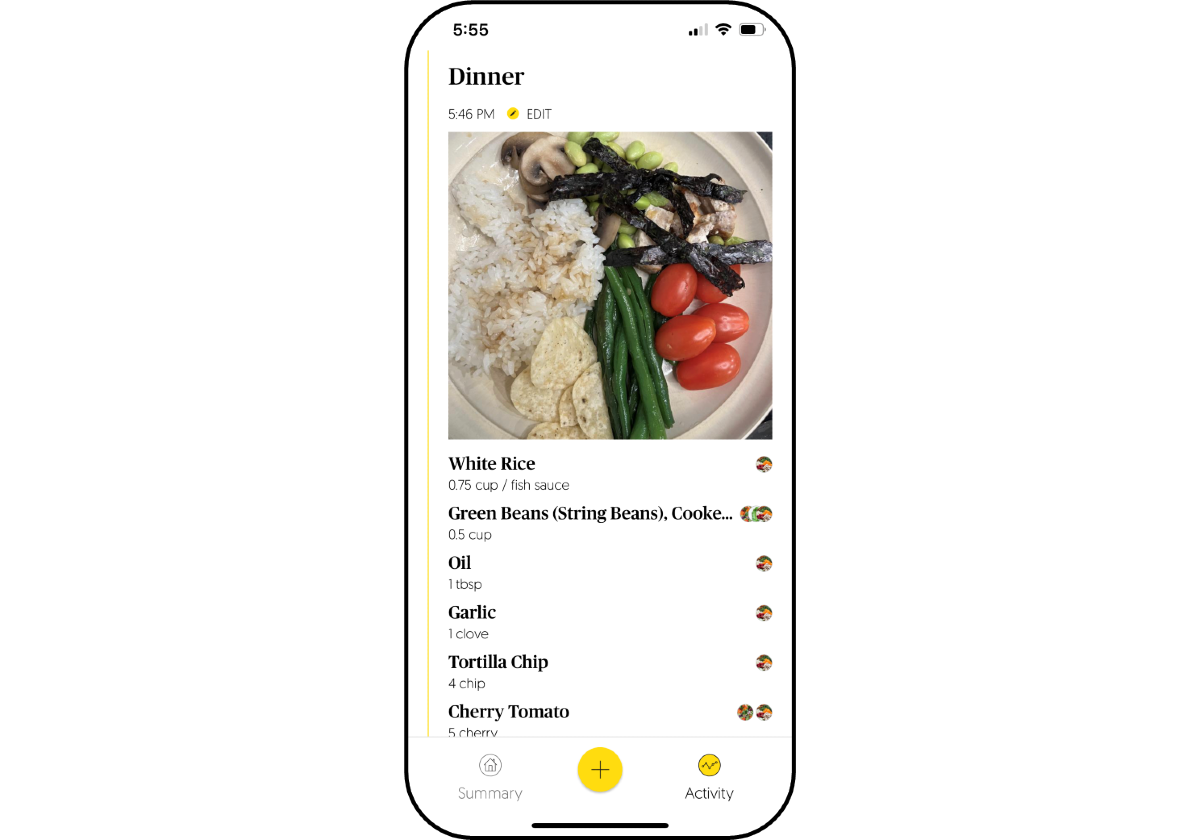
Example of the E4W app interface displaying food log results with modifications made after usability testing. E4W: Eating for Wellness.

Each patient participant completed 22 tasks across 6 scenarios (Figure 2). In cycle 1, a total of 23 (17%) out of 132 tasks could not be completed due to a critical issue (eg, technical errors requiring the app to be restarted and learnability issues related to editing a food item). Refinements to the app were made to make food editing more intuitive, and overall improvements to the proprietary algorithms, metadata processing, and connection to the cloud platform were made to improve efficiency. In cycle 2, a total of 4 (5%) out of 88 tasks could not be completed due to a critical issue. To address the critical learnability issue of users not knowing how to edit a food item, modifications to the user interface such as adding a pencil icon beside the food photo were made. In cycle 3, there was no task that was incomplete due to a critical issue. Only 5 (6%) out of 88 tasks were completed with a minor issue, and the remaining 83 (94%) tasks were successfully completed. As there were no critical issues identified in cycle 3, no further testing was conducted on the E4W app and testing was completed within 3 cycles (Figure 3).

AESs were completed by 13 (93%) out of 14 patient participants. Scores were positive with a mean overall satisfaction score of 4.1 (SD 0.9) out of 5. Mean scores in each successive cycle were as follows: cycle 1=4.0 (SD 0.9); cycle 2=4.3 (SD 0.8); and cycle 3=4.3 (SD 0.7). Scores across all individual areas (ease of use, easy to understand, helpfulness in identifying and documenting food intake, efficiency) were ≥4.0 out of 5. All patients (n=13) expressed a willingness to continue using E4W for a minimum period of 4 weeks, and 11 (85%) out of 13 patients expressed a preference to use E4W for food logging in the future instead of the traditional pen and paper methods.

Interviews revealed the following primary themes:

- Ease of use: patient participants found the E4W app simple and easy to use (AES score out of 5: mean 4.0, SD 0.7). The suggestions to improve ease of use were minor and included streamlining the process of deleting an erroneous food entry, streamlining navigation, and adding labels to certain icons.
- Efficiency: patient participants found the E4W app efficient (AES score out of 5: mean 4.0, SD 0.9). All the standardized tasks were collectively completed in less than 15 minutes. Aside from 2 participants whose image recognition took more than 3 minutes (which was subsequently resolved following an app update), all tasks and photo recognition were completed within 2 minutes or less.
- Functionality and features: patient participants rated the app as helpful in identifying their food (AES score out of 5: mean 4.2, SD 1.1) and helpful in documenting their food intake (AES score out of 5: mean 4.2, SD 0.9). The image recognition and goal-setting features were reported as the most important and the most liked feature of the app. There were mixed opinions on whether participants wanted calories or other micro- or macronutrient details for each food item to be visible to them (Yes: 7/14, 50%; No: 4/14, 29%; Unsure: 3/14, 21%). Most individuals (8/14, 57%) preferred to receive between 1 and 3 notifications per day (around mealtimes), 21% (3/14) of individuals preferred 4-6 notifications per day, and 14% (2/14) of individuals wanted ≥7 notifications per day as they worried they would otherwise forget to log their meals. These suggestions were considered when refining the app for Phase 2—pilot RCT of the study.
- Design aesthetics: app aesthetics were generally liked. The layout and general design of the app were described as “simple,” “clean,” and “well-organized.” Navigation was described as “easy” and “simple.” Several participants suggested adding a feature to customize the color scheme (eg, adding in a dark mode and allowing patients to tailor the color scheme to their liking) and adding animations or gamification features and felt that these would further engage adolescents in particular.

#### HCP Usability Testing

**Figure 4:**
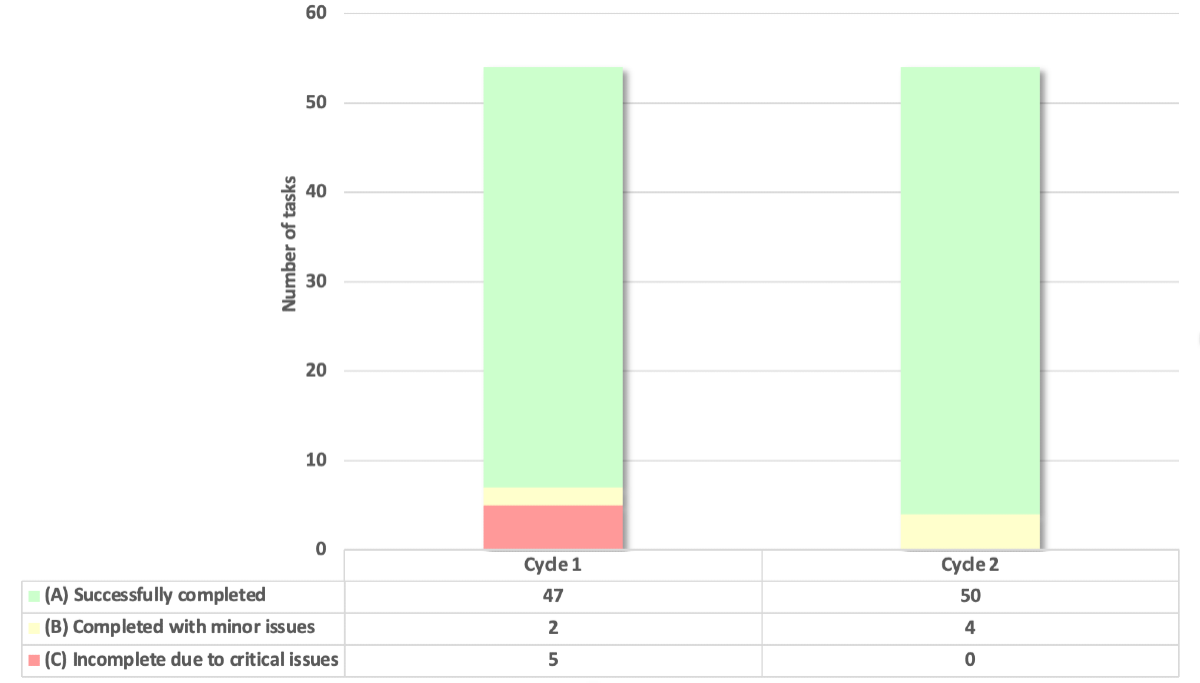
Phase-1 usability testing—HCP results. Errors and issues were tracked for each cycle and completion of tasks was classified into 1 of 3 categories: (A) successfully completed (shown in green), (B) completed with minor issues (shown in yellow), and (C) incomplete due to critical usability issues (shown in red). The figure shows the total tasks per category for the 2 cycles. HCP: health care provider.

Each HCP completed 27 tasks across 6 scenarios. Errors and issues were tracked for each cycle and classified into 1 of 3 categories (successfully completed, completed with minor issues, or incomplete due to critical usability issues; Figure 4).

In cycle 1, a total of 54 tasks were performed (2 HCPs each performed 27 tasks). In total, 5 (9%) tasks could not be completed due to a critical issue. Critical issues preventing the successful completion of tasks pertained to viewing the clinician report. This issue was fixed for the next cycle. In cycle 2, a total of 54 tasks were performed (2 HCPs each performed 27 tasks). No critical issues prevented the completion of tasks. Only 4 (7%) tasks were completed with a minor issue and the remaining 50 (93%) tasks were successfully completed. As there were no critical issues identified in cycle 2, no further testing was conducted.

AESs were completed by all HCPs. Scores were positive with a mean overall satisfaction score of 4.6 (SD 0.7) out of 5. Mean total scores across all individual areas were rated ≥4.5 out of 5. All HCPs (n=4) expressed a willingness to continue using E4W for the maximum period noted in the questionnaire (longer than 8 weeks), and all HCPs expressed a willingness to replace conventional recall-based 3-day food records with E4W and the Clinician Portal.

Interviews revealed the following primary themes:

- Ease of use: HCPs found the Clinician Portal simple and easy to use (AES score out of 5: mean 4.8, SD 0.5). Suggestions for improvement included minor layout suggestions for the Goals page and the Reports page. These modifications were made prior to beginning cycle-2 HCP usability testing.
- Efficiency: HCPs found the Clinician Portal to be efficient (AES score out of 5: mean 4.8, SD 0.5). HCPs recognized the Clinician Portal as a time-saving tool as it reduced the need to perform a manual dietary recall. HCPs were able to complete all the standardized tasks in less than 15 minutes, with most individual tasks taking only a few seconds.
- Functionality and features: HCPs rated the Clinician Portal as helpful in identifying areas of improvement for their patients (AES score out of 5: mean 4.5, SD 0.6). A total of 50% (2/4) of HCPs identified the “Food Log” page as the most important feature of the portal. The page displays all the meal photos categorized by day and meal with a detailed breakdown of food items per meal with corresponding portion sizes and carbohydrate content. Any suggested modifications were considered when refining the Clinician Portal for Phase 2—pilot RCT of the study.
- Design aesthetics: HCPs liked the general layout of the Clinician Portal and found it easy to navigate. Minor design changes (eg, having a brief description of the icon appear if you hovered over it) and layout changes to the Goals page were made following cycle 2.
- Future use: all HCPs (n=4) preferred to use E4W and the Clinician Portal in lieu of conventional recall-based 3-day food records with patients. All felt that the Clinician Portal would be useful in their day-to-day clinical assessments in the future.

### Phase 2—Feasibility Testing

#### Participant Characteristics

**Figure 5:**
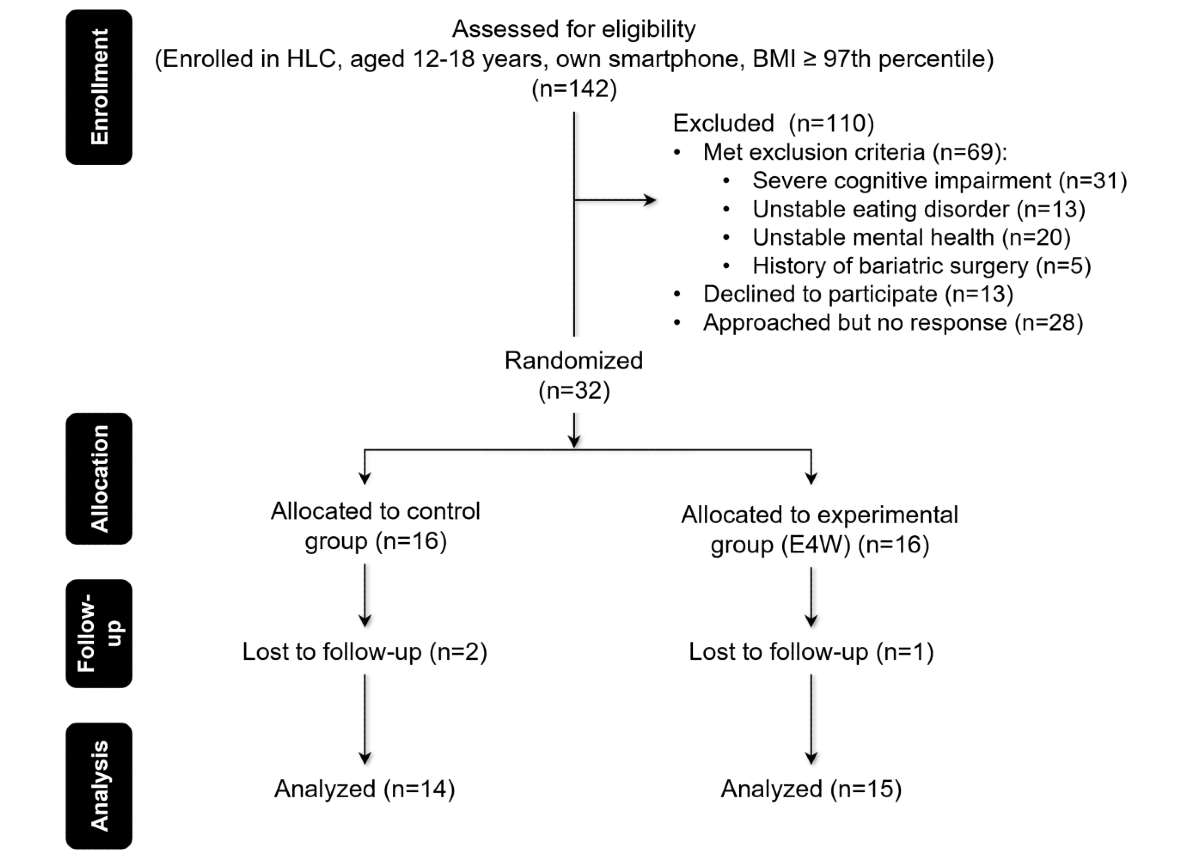
Phase-2 feasibility testing—CONSORT flow diagram. CONSORT: Consolidated Standards of Reporting Trials; E4W: Eating for Wellness; HLC: Healthy Living Clinic.

A total of 32 patient participants were recruited and enrolled in the study and randomized equally to the 2 arms of the study between August 2022 and April 2023. Dietitians had the ability to use the Clinician Portal for the E4W participants. A total of 29 participants completed the study (14 in the control group and 15 in the experimental group; Figure 5).

The demographics and clinical characteristics of participants are displayed in Table 2. There were no significant differences in baseline characteristics between the 2 groups (age: *P=*.051; sex: *P=*.48; ethnicity: *P*=.84; time in HLC: *P*=.18; BMI: *P*=.37; cardiometabolic comorbidities: *P=*.48; mental health condition: *P*=.47; history of food insecurity: *P*=.87; parental involvement: *P*=.89; type of phone: *P*≥.99; and use of social networking: *P*=.43; Table 2).

**Table 2:** Phase-2 feasibility testing—participant demographic and clinical characteristics (at baseline).

				All participants (n=32)		Control^a^ (n=16)		E4W^a,b^ (n=16)		*P* value^c^	
**Demographics**											
	**Age (years),** **mean (SD)**		15.3 (1.8)		14.6 (1.8)		15.9 (1.7)		.051		
	**Sex^d^, n (%)**										.48
		Male	16 (50)		9 (56)		7 (44)				
		Female	16 (50)		7 (44)		9 (56)				
	**Ethnicity, n (%)**										.84
		Black	8 (25)		4 (25)		4 (25)				
		Korean	1 (3)		1 (6)		- (0)				
		South Asian	2 (6)		2 (13)		- (0)				
		Chinese	2 (6)		1 (6)		1 (6)				
		Latin American	1 (3)		1 (6)		- (0)				
		West Asian	2 (6)		1 (6)		1 (6)				
		Filipino	1 (3)		- (0)		1 (6)				
		White	9 (28)		4 (25)		5 (31)				
		Other	6 (19)		2 (13)		4 (25)				
**Clinical characteristics**											
	**Time in HLC^e^** **(years),** **mean (SD)**		1.2 (1.5)		1.6 (1.4)		0.9 (1.5)		.18		
	**BMI (kg/m^2^),** **mean (SD)**		39.7 (7.6)		38.4 (8.7)		40.9 (6.4)		.37		
	**Comorbidities,** **n (%)**										
		Cardiometabolic^f^	16 (50)		7 (44)		9 (56)		.48		
		Mental health condition^g^	20 (63)		9 (56)		11 (69)		.47		
	**History of food insecurity,** **n (%)**		8 (25)		5 (31)		3 (19)		.87		
	**Parental involvement in supporting nutrition goals,** **n (%)**										.89
		Moderate-high	20 (69)		9 (64)		11 (73)				
		Absent-low	9 (31)		5 (36)		4 (27)				
**Technology use**											
	**Type of Phone,** **n (%)**										>.99
		Android	8 (28)		4 (29)		4 (27)				
		iPhone	21 (72)		10 (71)		11 (73)				
	**Use of social networking sites for nutritional information,** **n (%)**		9 (31)		3 (21)		6 (40)		.43		

^a^Two participants from the control group and 1 participant from the E4W group were lost to follow-up. These 3 participants therefore had missing data for the following characteristics: parental involvement in supporting nutrition goals, type of phone, and use of social networking sites for nutritional information.

^b^E4W: Eating for Wellness.

^c^*P* values represent the statistical baseline differences between the control and E4W groups. For continuous data, 2-sided independent *t* tests were used. For categorical variables, chi-square tests (for expected cell sizes >5) and Fisher exact tests were used.

^d^Patients reported both sex and gender. In total, 31 (97%) out of 32 patients expressed a gender that was consistent with their biological sex (cisgender). One patient preferred not to respond.

^e^HLC: Healthy Living Clinic.

^f^Cardiometabolic comorbidities included any one of the following: type 2 diabetes or medication-induced diabetes, dyslipidemia, hypertension, metabolic dysfunction–associated steatohepatitis, and polycystic ovary syndrome.

^g^Mental health conditions included a past or present diagnosis of a mental health condition such as depression, anxiety, obsessive-compulsive disorder, history of self-harm or suicidality, attention-deficit/hyperactivity disorder, or an eating disorder. Patients with unstable mental health conditions or eating disorders were excluded from the study. As such, eating disorders or mental health conditions represented here pertain to stable eating disorders or mental health conditions not felt to preclude assessment. These participants were deemed to be eligible to partake in the study by their HLC clinicians.

#### Primary Outcomes

##### Study Completion Rates

In total, 29 (91%) out of the 32 participants completed the study (control: 14/16, 88% completion, experimental: 15/16, 94% completion; *P*=.99).

##### Technical Difficulties

There were 4 main types of technical errors encountered by the participants: failure to receive automatic push notifications (10/15, 67%), failure to receive customized dietitian tips (11/15, 73%), app crashes (5/15, 33%), and deletion of photos taken (3/15, 20%).

On November 30, 2022, the app was moved to a new, more stable platform, and a new version was released. There were no further instances of app crashes or deletion of photos following this release. Unfortunately, there were still some participants who reported not having received notifications, despite having been delivered by the app server. On further investigation, it appears that the notifications were blocked by the user’s phone due to an overall notification setting on their device.

##### Patient Engagement

To assess patient engagement, the average number of photos taken per day per participant was calculated. Patients in the control group took an average of 0.9 (SD 0.7) photos per day while those in the experimental group took an average of 1.7 (SD 1.9) photos per day (*P*=.14). However, a subset of the experimental group exhibited much higher levels of engagement than their control counterparts. There was 1 participant outlier in the experimental group who displayed high use of the app with 195 photos taken (an average of 6.1 photos per day) during the study period. No outliers were identified in the control group. To better understand the different levels of engagement for the app users, the total number of photos taken throughout the study period was broken down into 3 general categories (no to little engagement, n=4: participants who took less than 10 photos throughout the study period; moderate engagement, n=6: participants who took between 10 and 45 photos; and high engagement, n=5: participants who took greater than 45 photos throughout the study period). One-third (2/6, 33%) of the participants with moderate engagement felt that their dietary habits had changed during the study due to the use of the app. Participants in the high engagement category each took ≥79 photos throughout the study period, with 80% (4/5) of them taking >100 photos. During postintervention interviews, 80% (4/5) of the participants with high engagement felt that their dietary habits had changed during the study due to the use of the app.

##### Patient Acceptability and Satisfaction

The overall mean AES score was 4.1 (SD 0.7) out of 5. Generally, the scores were similar between period 1 and period 2 (pre and post November 30, 2022, technical fixes). A total of 93% (13/14) of patients indicated they would prefer to continue using E4W to log food intake over traditional methods.

#### Secondary Outcomes

##### Dietary Goals

The average number of goals completed throughout the study period was 3.3 (SD 1.5) goals for the experimental group and 4.3 (SD 1.7) goals for the control group. Overall, there was no statistically significant difference (*P*=.12) in successful goal attainment between the 2 groups.

##### Change in Dietary Intake

Overall, participants using E4W showed a statistically significant increase in their recorded daily caloric intake between baseline and final visit compared to the control group (mean +469, SD 754 kcal vs mean –152, SD 635 kcal; *P*=.04; Table 3) and a statistically significant increase in recorded daily saturated fat intake compared to the control group (*P*=.01; Table 3).

**Table 3:** Phase 2 feasibility testing—change in daily dietary intake from baseline to final visit.

Outcomes	Baseline			Final			Change		
	Control (n=13)	E4W^a^ (n=10)	Control (n=13)		E4W (n=10)	Control (n=13)		E4W (n=10)	*P* value
Energy (kcal), mean (SD)	1642 (421)	1539 (279)	1490 (394)		2007 (815)	–152 (635)		469 (754)	.04
Sugars (g), mean (SD)	52.5 (21)	66.8 (47)	62.4 (26)		77.7 (49)	9.9 (44)		10.9 (60)	.96
Fiber (g), mean (SD)	14.2 (5)	11.6 (6)	15.6 (5)		14.2 (6)	1.3 (6)		2.6 (4)	.58
Protein (g), mean (SD)	78.5 (27)	81.6 (19)	63.7 (20)		83.2 (29)	–14.8 (25)		1.7 (24)	.13
Unsaturated fat (g), mean (SD)	43.2 (18)	35.9 (11)	38.2 (19)		53.9 (30)	–5.0 (25)		17.9 (30)	.06
Saturated fat (g), mean (SD)	21.2 (7)	18.2 (7)	16.7 (5)		26.7 (11)	–4.5 (8)		8.5 (14)	.01
Fruits and vegetables (cup equivalents), mean (SD)	2.9 (1)	2.3 (1)	2.7 (1)		2.3 (2)	–0.3 (1)		0.0 (1)	.57

^a^E4W: Eating for Wellness.

Changes in all other macronutrients (total sugar: *P*=.96; fiber: *P*=.58; protein *P*=.13; and unsaturated fat: *P*=.06) and total fruits and vegetable intake (cup equivalents; *P*=.57) from baseline to final visit between the 2 groups were not statistically significant.

## Discussion

### Principal Findings

Accurate dietary assessments in adolescents with obesity remain a challenge due in part to the low completion rate and accuracy of traditional food logging methods [5-7]. To date, no studies have explored the potential of image-recognition apps to make dietary assessments in adolescents with obesity. In this study, we performed usability and feasibility testing of E4W, an image-recognition app that offers a promising, user-friendly, efficient, and novel method to log food intake while benefiting from personalized self-monitoring support from a dietitian.

Our findings from the usability testing revealed that E4W and the Clinician Portal were both easy to use, well-liked, and efficient. Both adolescents and HCPs successfully completed standardized scenario-based tasks in a timely manner with high efficiency and satisfaction scores, with the majority reporting a preference to use E4W to log food intake instead of conventional 3-day recall methods. Critical usability issues identified were addressed. Changes made included improvements to the proprietary algorithms, metadata processing, and user interface to make food editing more intuitive. The refined app and portal were then used for phase 2 of the study.

In phase-2 feasibility testing, the app met 2 of the 4 criteria for implementation success (study completion rate and patient acceptability and satisfaction). Unfortunately, participants enrolled earlier in the pilot RCT experienced major technical difficulties around receiving app notifications. This issue likely impacted engagement and may have also dampened acceptability and satisfaction scores due to the lack of reminders and reduced interaction. Despite these challenges, overall AES scores still remained highly positive, and improvements and fixes have been implemented to enhance the stability of the app.

Although this study was not designed to assess dietary choices as a key outcome, we did examine the available data. Participants using E4W did show statistically significant increases in their recorded daily caloric intake (*P*=.04) and recorded daily saturated fat intake (*P*=.01) between baseline and final visit compared to the control group. Changes in other specific macronutrients were not significant. It is important to note that traditional 3-day food records were used as the primary method to measure dietary intake for both the control and experimental groups. Caution should therefore be exercised with the interpretation of these results, as overall adherence to traditional 3-day food records was quite poor in both groups and resulted in missing data for the initial and final visits of both groups. Results in both groups were likely affected by errors, biases, and inaccuracies inherent in traditional 3-day food records.

E4W did not significantly impact dietary goal achievement compared to control (*P*=.12). Goals were set using the clinic’s existing approach to avoid deviation from standardized care, tailoring them individually. This approach led to nonstandardized goals across groups. This created a potential bias for more challenging goals to be assigned to the experimental group, assuming greater motivation or engagement with the app, and potentially reducing goal attainment. Interdietitian variability also influenced goal types.

Now that E4W has addressed usability and feasibility issues, it will be important to conduct a rigorous study of its impact on adolescent healthy dietary choices supported by dietitians.

### Comparison to Previous Work

Recent surveys have called for the use of technological tools to aid HCPs in their dietary assessments and described conventional recalls as time-consuming and sometimes unreliable [7]. The positive results from this usability study demonstrate that E4W and the Clinician Portal may be important tools to address this need.

There have been limited published studies assessing the usability of mobile health interventions for adolescents with obesity. According to a recent narrative review summarizing usability and engagement testing of mobile health apps in children with obesity [45], a total of 6 studies [46-51] conducted across the United States, Ireland, Switzerland, and Italy performed usability testing. Unlike E4W, 3 of the studies [47,49,51] involved tasks that required parental supervision or completion of the task by parents themselves, demonstrating the difficulties with making suitable yet effective apps for adolescents themselves. App features ranged from text-based coaching to physical activity and diet monitoring, while one focused on culinary nutrition education [49]. None of these studies used image-recognition dietary assessment apps. Those apps that incorporated dietary monitoring required users to enter their food intake either by text entry or selection from a menu.

Other relevant usability and feasibility studies include a validation study of a similar image-based dietary assessment app, Keenoa, conducted in healthy adults [52]; however, usability and future use scores for this app were lower than those for E4W. The impact of FRANI (Food Recognition Assistance and Nudging Insights, another image-based dietary assessment app) [53-55] on food choices and diet quality in female adolescents in Vietnam was also conducted in a pilot RCT comparing the gamified FRANI to a FRANI version limited to dietary assessment. The study showed that the use of the app was feasible in this population with high adherence. The use of the gamified FRANI was associated with a significantly higher Eat-Lancet Diet Score [56]. These apps, however, were not designed specifically for adolescents with obesity.

The potential of pediatric digital health interventions to affect dietary intake has been demonstrated for outcomes such as self-reported decrease in sugary beverages, lower carbohydrate intake, increased fresh fruit consumption, and a decrease in juice intake [57]. However, none of these interventions used an app with image recognition of dietary intake. Future studies will evaluate if this technology, as part of E4W, leads to more pronounced changes in dietary intake.

Limitations of existing nutrition apps include an emphasis on calorie counting and weight loss [18], which are not in line with the goals of pediatric obesity management programs. These conflicting designs could trigger negative thinking patterns in adolescent users, such as obsession over calories, feelings of low self-worth and guilt, and compensatory behavior such as overexercising or purging and even development of eating disorders [58]. To address these concerns, E4W encourages healthy eating behaviors with the support of a dietitian and without nutritional information being visible to the patient.

### Strengths and Limitations

This study offered several strengths. Although our sample size was small, the study incorporated a population with diverse ethnic backgrounds, sex, age, smartphone operating system, and length of time followed in a pediatric weight management program. Capturing a diverse multiethnic population was important given the range of cuisines, eating habits, and schedules across cultural backgrounds to ensure a more complete and generalizable assessment of functionalities and features. Another strength of the study was the patient-centered and end user approach to developing and refining E4W and the Clinician Portal, as well as qualitative data acquisition.

A limitation of the study was that it was conducted in a single tertiary pediatric center. However, as it is a tertiary center with a large referral base from all across Ontario, the clinic generally encompasses a diverse multiethnic patient population, which was reflected in our sample. Furthermore, although pediatric weight management clinics in a tertiary center may have a more medically complex patient population than a community site, the complexity created a more challenging testing environment. Therefore, it is possible that success in this study environment could be generalizable to less complex patient populations.

Second, usability testing was conducted in a controlled testing environment with prestandardized tasks, and therefore, may not be reflective of real-life use of the app and the Clinician Portal. Furthermore, as the study was conducted during a period with varying levels of social-distancing restriction during the pandemic, many of the testing sessions and dietitian visits were conducted virtually. Although this was a limitation to the study in some ways, it provided a unique opportunity to perform usability testing in the patient’s home environment, using their own smartphone devices and using food from their home environment and respective cultures—rendering the usability testing more realistic and reflective of real-world use of the app.

Finally, due to the technical difficulties participants experienced with automatic push notifications and dietitian tips, appropriate assessment of the usability of this function was limited. Although these issues have now been resolved and led to an improved and stable app and a more efficient and effective means of identifying and fixing technical issues in the future, further testing in this area is needed.

### Conclusions

In conclusion, we tested a novel approach to assessing dietary intake in adolescents with obesity and presented evidence supporting the usability of E4W and its associated Clinician Portal for HCPs in a pediatric weight management program. Overall, the app was highly rated, easy, and efficient to use, despite some early technical difficulties at the start of the trial.

Those individuals who displayed high levels of engagement also expressed high degrees of satisfaction and self-perceived behavioral change with the use of E4W. This offers encouragement that the use of E4W may promote improved dietary habits in users who show higher engagement with the app. Further work is needed to identify how to best implement these technologies into patient care in order to support adolescents in developing healthier eating behaviors.

## Appendix

Multimedia Appendix 1 CONSORT-eHEALTH checklist (V 1.6.1).

## Abbreviations

AES: Acceptability E-Scale
CONSORT-C: Consolidated Standards of Reporting Trials–Children
E4W: Eating for Wellness
FRANI: Food Recognition Assistance and Nudging Insights
HCP: health care provider
HLC: Healthy Living Clinic
RCT: randomized controlled trial
REDCap: Research Electronic Data Capture
SMART: Specific Measurable Attainable Realistic Time-Bound
SPIRIT-C: Standard Protocol Items for Randomized Trials–Children

## Data Availability

The datasets generated during or analyzed during this study are available from the corresponding author upon reasonable request.
